# Drug Use Evaluation of Beta-Blockers in Medical Wards of Nedjo General Hospital, Western Ethiopia

**DOI:** 10.1155/2020/2509875

**Published:** 2020-05-22

**Authors:** Ginenus Fekadu, Firomsa Bekele, Kumera Bekele, Sagni Hanbisa, Gemechis Belay, Mudasir Maqbool

**Affiliations:** ^1^Department of Pharmacy, Institute of Health Science, Wollega University, Nekemte, Ethiopia; ^2^Department of Pharmacy, College of Health Science, Mettu University, Mettu, Ethiopia; ^3^Department of Nursing, College of Health Science, Selale University, Fiche, Ethiopia; ^4^Department of Pharmaceutical Sciences, University of Kashmir, Hazratbal Srinagar, 190006 Jammu and Kashmir, India

## Abstract

**Introduction:**

Beta-blocker use evaluation is a performance method that focuses on the evaluation of beta-blocker use processes to achieve optimal patient outcomes. Several studies conducted in different hospitals revealed a high incidence of inappropriate prescription of beta-blockers among hospitalized patients. Therefore, it is important to identify inappropriate beta-blocker prescribing since they may increase the risk of hospitalizations. Despite this, there was no study conducted related to drug use evaluation of beta-blockers in Nedjo general hospital (NGH). Thus, this study was aimed at assessing the use evaluation of beta-blockers in medical wards of NGH.

**Methods:**

A retrospective cross-sectional study was conducted at medical wards of NGH from January 1, 2016, to December 31, 2017.

**Results:**

Out of the total of 149 medical record of patients that contains beta-blockers, 84 (56.37%) were males and about one-third (31.54%) of the patients ages were between 41 and 50 years. Propranolol was the most commonly prescribed beta-blocker (62.76%), and 94.56% of beta-blockers were prescribed with correct indication. There were about 51%, 46.31%, 64.43%, and 46.98% of beta-blockers prescribed with the correct dose, duration, frequency, and route of administration, respectively. Regarding the routes of administration, 70 (46.98%) of them were prescribed with the correct route. Most drugs interacting were propranolol with cimetidine 26 (68.42%), and the most frequent condition for which beta-blockers were prescribed was hypertension (32.89%).

**Conclusion:**

Overall, there was an inappropriate use of beta-blockers in terms of dosage and durations. So, prescribers of NGH should strictly adhere to the national treatment guideline when prescribing medications. Additionally, drug information centers have proved useful and effective in promoting rational drug use. Hence, it should be recommended for general use.

## 1. Introduction

The potential inappropriate uses of drugs are becoming a concern worldwide with their increment in quantity and variety. It is associated with health risks to the patient and financial crisis to the health facilities and patients [1–3]. Beta-blockers are commonly used in family practice to manage a wide variety of chronic diseases, including hypertension. They are a distinct group of antihypertensive agent which has different efficacy depending on demographic groups and other comorbid conditions [4–6]. Trials have evaluated the effects of various beta-blockers on outcomes in patients with hypertension [5, 7]. Acute beta-blocker administration may reduce the presence and severity of myocardial perfusion defects with dipyridamole stress [8].

Several randomized clinical trials have established the beneficial effects of the beta-blockers in patients with chronic systolic heart failure and normal or mildly reduced renal function. However, limited evidence exists about the safety and efficacy of these agents in patients with heart failure with reduced ejection fraction and end-stage renal disease [9, 10].

Few studies suggested that coronary flow reserve will be measured using positron emission tomography (PET) is improved in steno sis-dependent segments of the myocardium during long-term beta-blocker treatment; thereby, beta-blockers may decrease the contrast between ischemic and nonischemic myocardium during hyperemia induced by dipyridamole [11, 12]. Additionally, beta-blockers are a cornerstone treatment of patients with heart failure. Randomized trials with carvedilol, metoprolol, bisoprolol, and nebivolol showed that beta-blockers reduce morbidity and mortality in heart failure patients [11, 13].

Although beta-adrenergic blockers can significantly reduce mortality after a myocardial infarction, these agents are prescribed to only a minority of patients. Underutilization of beta-blockers may be attributed, in part, to fear of adverse effects, especially in the elderly and in patients with concomitant disorders such as diabetes or heart failure [12, 14, 15]. The American Heart Association and the American College of Cardiology emphasize the importance of beta-blockade in their current treatment guidelines for myocardial infarction. Based on these guidelines, the Health Care Financing Administration (HCFA) and the National Coalition for Quality Assurance (NCQA) have identified beta-blocker therapy after myocardial infarction as a critical marker for quality of care [16]. Besides, emerging data on the safety and benefits of beta-blockers in patients with congestive heart failure have led to a reconsideration of the use of beta-blockers under circumstances in which these drugs were previously considered contraindicated [17].

Despite the benefits of these agents, data from health services research studies show that beta-blockers are often not used in patients who have had a myocardial infarction [14, 18]. United States' guidelines are less aggressive in their support of perioperative beta-blockers, and it found that beta-blocker use increased the odds of having an acute coronary event [18, 19].

Due to several reasons, hospitalized patients are likely to receive more medications than those treated as outpatients [20]. In the general population, it is estimated that approximately 5 to 10% of all hospital admissions are drug-related, and about 22% of patients are discharged with inappropriate drugs. About 28% of emergency department visits are drug-related [21].

It is important to identify inappropriate beta-blocker prescribing since it may increase the risk of hospitalizations. It has been estimated that up to 30% of hospital admissions in the older population may be related to medication problems [22].

According to the study conducted in Jimma University, specialized hospital beta-blockers were frequently inappropriately prescribed. Atenolol was prescribed inappropriately for almost half of the patients [23]. Pharmacist-led medication reviews in patients' admitted to the medical ward led to medication changes in more than three-forth of the patients [24].

Good adherence to pharmacologic treatment guidelines for beta-blocker with prescription of at least 50% of recommended dosages was associated with better clinical outcomes, and continuing global educational initiatives are needed to emphasize the importance of guideline recommendations for optimizing drug therapy [25]. Despite this, in our setup, there were no guidelines and a predetermined protocol to prescribe beta-blockers.

Inappropriate use of drugs which usually arises from multiple prescribing and dispensing errors is a common problem, especially in developing countries, substantially contributing to deleterious effects on health and economic burden [26, 27]. Several studies conducted in different hospital showed a high incidence of inappropriate prescription of beta-blockers among hospitalized patients. But there were no studies conducted related to drug use evaluation of beta-blockers in NGH. Thus, this study was aimed at assessing the use evaluation of beta-blockers in medical wards of NGH.

## 2. Methods and Materials

### 2.1. Study Setting and Period

The study was conducted at NGH located at 501 km to western direction from Addis Ababa. A retrospective cross-sectional study was conducted from January 1, 2016, to December 31, 2017. NGH has different departments and wards like outpatient department, medical, gynecology and obstetrics, pediatrics, and surgical wards. It delivers diversified health services and clinics including the emergency services, eye clinic, mother and child health, psychiatry clinic, laboratory, X-ray, and follow-up of chronic diseases like tuberculosis (TB), diabetes mellitus (DM), and HIV/AIDS as well as outpatient and inpatient pharmacies.

### 2.2. Study Participants and Eligibility Criteria

All patients admitted to the medical wards of NGH taking at least one beta-blocker with or without other drugs during the study period having full information were included. Whereas, patient cards with incomplete information were excluded.

### 2.3. Sample Size and Sampling Technique

The minimum sample size was estimated by the single population formula.

(1) $$\begin{array}{c} \begin{array}{c} N=\frac{{\left(Z\alpha /2\right)}^{2}p\left(1-q\right)}{d^{2}}\\ =\frac{{\left(1.96\right)}^{2}0.5\times 0.5}{{\left(0.05\right)}^{2}}=384 \end{array} \end{array}$$

where *N* is sample size, *P* is the proportion of the evaluation of beta-blockers (50%), *Z* is the standardized normal distribution value at 95% CI: 1.96, and *d* is the margin of the sample error tolerated (5%).

But in medical wards of NGH during 2 years of the study period, the total number of patients taking beta-blockers were 243. Since the total number of populations was less than 10,000, the minimum final sample size was calculated by using the adjustment reduction formula. The corrected sample size, using the correction formula was 149. The samples were drawn by using a systematic random sampling method.

### 2.4. Data Collection Process and Management

Data collection format was developed based on previous literature. The format contained patient information (presence of sex, age, and card number), clinical information, and pattern of beta-blocker. Each case from the patient medication records was evaluated against the up to date information and standard treatment guideline (STG) of Ethiopia for indication, dosage, frequency, and duration of beta-blocker therapy. Before starting actual data collection, small scale data collection was done on the data collection format for having full required information of the study and to maintain the quality of the study. Dosage regimens (dose, frequency, and duration), indications, drug interaction, and contraindication were dependent variables.

### 2.5. Data Processing and Analysis

The data was entered into the computer using the EPI-manager 4.0.2 software. Data checking and cleaning were done by a principal investigator on a daily basis during collection before the actual analysis. The analysis was done using the Statistical Package for Social Sciences (SPSS) 24. Descriptive data was generated and placed in terms of frequency and percentage. Data was presented by using tables.

### 2.6. Operational Definitions

#### 2.6.1. Beta-Blocker Drugs

Beta-blocker drugs are adrenergic receptor blockers that are used for the treatment of several diseases such as hypertension, ischemic heart disease, congestive heart failure, and certain arrhythmia [28].

#### 2.6.2. Appropriate Drug

The selection of drugs is based on efficacy, safety, suitability, and cost considerations or when the drug prescribed for the diagnosis made is compliant in terms of indication, dose, frequency, and duration of therapy [3, 29].

#### 2.6.3. Compliance

Compliance is the degree to which patients adhere to medical advice and take drugs as indicated [3, 29].

#### 2.6.4. Drug Use Evaluation

Drug use evaluation is a system of ongoing, systematic criteria-based evaluation of drug use that will help ensure that medicines are used appropriately at the individual patient level [1, 18].

#### 2.6.5. Inappropriate Use

Inappropriate use is failure to prescribe as per clinical guidelines [29].

#### 2.6.6. Prophylactic Treatment

Prophylactic treatment is administration of drugs to prevent possible infection before its occurrence [4, 30].

## 3. Result

### 3.1. Sociodemographic Distribution of Patients

A total of 149 patients' medical record which contains beta-blockers was reviewed; from these, males were 84 and 65 patients were females. The majority, 47 (31.54%) of the patients, were between 41 and 50 years (Table 1).

### 3.2. Categories and Indications of Beta-Blockers

The most commonly prescribed beta-blockers were propranolol, 93 (62.76%), and atenolol, 56 (37.24%). From the study, most and least frequent diseases for which beta-blockers are prescribed were hypertension (32.89%) and thyrotoxicosis (4.70%), respectively. The maximum combination was propranolol with cimetidine and hydrochlorothiazide, 24 (33.33%), and the minimum combination was propranolol and cimetidine which accounts 2 (2.78%) (Table 2).

From the study, 141 (94.56%) of beta-blockers used were an appropriate indication. Major indication, 66 (44.23%), of beta-blockers was stage two hypertension, and minor indication, 11 (7.38%), was thyrotoxicosis. From the study, 6 (4.1%) of beta-blockers used were incorrect indication with regard to stage one hypertension, and 2 (1.34%) of beta-blockers used were incorrect indication with regard to stage two hypertension with asthma or type one diabetes mellitus (Table 3).

### 3.3. Dosage Regimen and Route of Administration of Beta-Blockers

From the study, 76 (51%) of beta-blockers used were containing the correct dose, 24 (16.11%) underdose, and 49 (32.89%) overdose. About 69 (46.31%) of beta-blockers were prescribed with correct duration, 21 (14.09%) of beta-blockers short duration, and 59 (39.60%) with a long duration. About 96 (64.43%) of beta-blockers were correct frequency, and 53 (35.57%) of them were incorrect frequency. Regarding the routes of administration, 70 (46.98%) of them were prescribed with the correct route, and 79 (53.02%) of them were prescribed with incorrect routes of administration (Table 4).

### 3.4. Drug Interaction and Contraindication of Beta-Blockers

During the comorbid conditions of hypertension and heart failure, prescribing atenolol for hypertension and digoxin for congested heart failure can increase the risk of Bradyarrhythmias. From the study, the majority of the drug interactions were between propranolol and cimetidine 26 (68.42%) (Table 5).

From the study, the duration of beta-blockers that patients receive was 15-30 days (46.99%) followed by 7-14 days, <7 days, and >30 days with 68 (45.65%), 6 (4%), and 5 (3.36%), respectively. From the study, 12 (38.71%) of atenolol at high dose and 19 (61.29%) of propranolol were contraindicated in comorbid illness of hypertension with asthma or type 1 diabetes mellitus.

## 4. Discussion

The rational use of drug requires that patient receives medication appropriate to their clinical needs in the dose that meet their own individual requirements for an adequate period and at an affordable cost to them [30, 31]. There were 4.1% of incorrect indication regarding stage 1 hypertension and 1.34% of incorrect indication with regard to stage 2 hypertension with asthma or type 1 diabetes mellitus due to the use of propranolol or atenolol at high dose which may lead to treatment failure or other unwanted effects, but this result was higher when compared with the previous study due to substandard of the hospital [3]. Similarly, the finding of Saudi Arabia showed a slightly high rate of propanol misuse [32].

The study revealed that 94.56% of the indications of beta-blockers were correctly used as compared to the DUE criteria of a threshold of 95%. So, there were appropriate uses of beta-blockers pertaining to indication. From the study, it was indicated that 51% of beta-blockers are with the correct dose, 46.31% of correct duration, and 64.43% of correct frequency with 46.98% of correct route of administration, but this result was lower when compared with the research done in the United States of America due to the lack of rational drug use [33]. The study also revealed that 16.11% of beta-blockers contain underdose,14.09% of short duration, 32.89% of overdose, 39.60% of long duration, 35.57% of incorrect frequency, and 53.02% of incorrect route of administration, but this result was higher when compared with the research done on the USA due to the lack of updated information in the guideline [34]. Similar findings were reported in Lebanon in which beta-blockers were the most commonly prescribed class of medication requiring dosage adjustment [35].

The problem of under dose and short duration leads to the reduction of the effectiveness of treatment which leads to treatment failure and different complications. From the study, it was indicated that 32.89% and 39.60% of beta-blockers were overdose and long duration, respectively, that lead to toxicity. This study also indicated that about 35.57% beta-blockers were incorrect frequency which may increase the possibility of toxicity and make administration difficult. It was indicated that atenolol acute toxicity in women lead to severe sinus bradycardia, hypotension, and marked hypoglycemic occurrence. The propranolol acute toxicity leads to bradycardia, severe hypotension (which may result in peripheral cyanosis), loss of consciousness, and seizures. This study was similar to the previous findings which revealed that there was a high incidence of over dosing [36, 37].

The finding of this research was different from cross-sectional study done in the intensive care unit of Jimma University Specialized Hospital which showed that the prevalence of medication prescribing error was 52.5%; the common prescribing error was using the wrong combination of drugs (25.7%), wrong frequency (15.5%), and wrong dose (15.10%) [38]. From the findings, it is evident that the result of the dosage regimen was lower in the DUE criteria of the threshold of 95% for beta-blockers. This may be due to a lack of adequate drug information or pharmaceutical care service. Drug interactions are some of the commonest causes of adverse effects. When two or more drugs are administered to the patients, they may act independently of each other or interact with each other. Interaction may increase or decrease the effect of drugs or the concentration of drugs and may cause unexpected toxicity or modify the effect of the drugs.

The study revealed that a maximum of beta-blockers contained one or more potential interacting drugs with atenolol with digoxin (7.89%), cimetidine with propranolol (68.42%), and propranolol with aluminum-containing antacid (23.68%). Drug interaction results were different from the DUE criteria of a threshold of 90% for beta-blockers; this difference may be due to the practice of prescribing multiple drugs by the prescribers. An adequate monitoring plan should be in place whenever we use a combination of atenolol and digoxin since the combination of both drugs may increase the risk of Bradyarrhythmias. Monitoring was also needed during a combination of aluminum-containing antacid and propranolol since aluminum-containing antacids may prevent proper absorption of propranolol. Other monitoring was also needed during a combination of propranolol with cimetidine since it increases steady-state plasma levels of propranolol and reduction in the liver first-pass elimination of propranolol [29].

Prescribing 61.29% propranolol and 38.71% atenolol at high dose is contraindicated in patients with stage 2 hypertension with asthma or type 1 diabetes mellitus with management of asthma or type 1 diabetes mellitus, but this result was higher when compared with the research done in Indonesia due to the lack of applicable drug information center [39].

### 4.1. Limitation of the Study

The study was cross-sectional so that the causal effect relationship was not determined. Additionally, it was not a multicenter study with a relatively small size. Therefore, generalization should be made cautiously.

## 5. Conclusion

The majority of beta-blockers were correctly indicated and fulfill the appropriate DUE criteria. So, there was an appropriate use of beta-blockers pertaining to indication. There were problems of the dose and duration of beta-blockers. So, prescribers of NGH should strictly adhere to guidelines when prescribing medications and activities like problem-based training in pharmacotherapy. A targeted continuing education and drug information center have proved useful and effective in promoting rational drug use and should be recommended for general use. We recommend further large population and longitudinal studies to assess drug use evaluation of beta-blockers over the period.

## Figures and Tables

**Table 1:** Socio-demographic distribution of patients taking beta-blockers in the medical wards of NGH within 2 years.

Age (in years)	Male		Female		Total	Percentage
	*n*	%	*n*	%	*n*	%
18-30	15	17.86%	13	20%	28	18.79%
31-40	16	19.05%	15	23.08%	31	20.81%
41-50	25	29.76%	22	33.84%	47	31.54%
>51	28	33.33%	15	23.08%	43	28.86%
Total	84	100%	65	100%	149	100%

**Table 2:** Frequently combined drugs with beta-blockers in the medical wards of NGH within 2 years.

Drugs combined with beta-blockers	Frequency (*n*)	Percentage (%)
Propranolol+cimetidine	2	2.78%
Propranolol+cimetidine+hydrochlorothiazide	24	33.33%
Propranolol+aluminum-containing antacid	9	12.50%
Hydrochlorothiazide+atenolol	4	5.56%
Hydrochlorothiazide+propranolol+aluminum-containing antacid	8	11.11%
Hydrochlorothiazide+atenolol+digoxin	12	16.67%
Atenolol+digoxin	3	4.17%
Hydrochlorothiazide+propranolol	10	13.89%
Subtotal	72	100%

**Table 3:** Reasons and indications of beta-blockers used in the medical wards of NGH within 2 years.

Variable	Reasons for use (disease)	Frequency (*n*)	Percentage (%)
Correct indication	Stage 2 hypertension	66	44.23%
	Cirrhosis	17	11.41%
	Atrial fibrillation	19	12.75%
	Migraine headache	28	18.79%
	Thyrotoxicosis	11	7.38%
	Subtotal	141	94.56

Incorrect indication	Stage 1 hypertension	6	4.1%
	Stage 2 hypertension with asthma or type 1 diabetes mellitus	2	1.34%
	Subtotal	8	5.34%

**Table 4:** Dosage regimens of beta-blockers used in the medical wards of NGH within 2 years.

Dosage regimen	Variables	Frequency (*n*)	Percentage (%)
Dose	Correct dose	76	51%
	Underdose	24	16.11%
	Overdose	49	32.89%

Duration	Correct duration	69	46.31%
	Short duration	21	14.09%
	Long duration	59	39.60%

Frequency	Correct frequency	96	64.43%
	Incorrect frequency	53	35.57%

**Table 5:** Potential drug interaction between some concomitantly prescribed drugs with beta-blockers in the medical wards of NGH within 2 years.

Drug interactions	Frequency (*n*)	Percentage (%)	Reasons for interaction
Propranolol+cimetidine	26	68.42%	Reduction in liver first-pass elimination of propranolol
Propranolol+aluminum-containing antacid	9	23.68%	Aluminum-containing antacids may prevent proper absorption of propranolol
Atenolol+digoxin	3	7.89%	Increase the risk of Bradyarrhythmias
Subtotal	38	100%	

## Data Availability

The data used to support the findings of this study are available from the corresponding author upon request.

## Abbreviations

CI: Confidence interval
DUE: Drug use evaluation
NGH: Nedjo General Hospital
SPSS: Statistical Package for Social Sciences
STG: Standard treatment guideline
WHO: World Health Organization.
